# Predefined and data driven CT densitometric features predict critical illness and hospital length of stay in COVID-19 patients

**DOI:** 10.1038/s41598-022-12311-4

**Published:** 2022-05-17

**Authors:** Tamar Shalmon, Pascal Salazar, Miho Horie, Kate Hanneman, Mini Pakkal, Vahid Anwari, Jennifer Fratesi

**Affiliations:** 1 Joint Department of Medical Imaging, University of Toronto, Toronto, ON Canada; 2 University Health Network, Toronto General Hospital, 200 Elizabeth St, Toronto, ON M5G 2C4 Canada; 3 Canon Medical, Minnetonka, MN USA

**Keywords:** Health care, Risk factors, Signs and symptoms, Engineering

## Abstract

The aim of this study was to compare whole lung CT density histograms to predict critical illness outcome and hospital length of stay in a cohort of 80 COVID-19 patients. CT chest images on segmented lungs were retrospectively analyzed. Functional Principal Component Analysis (FPCA) was used to find the main modes of variations on CT density histograms. CT density features, the CT severity score, the COVID-GRAM score and the patient clinical data were assessed for predicting the patient outcome using logistic regression models and survival analysis. ROC analysis predictors of critically ill status: 87.5th percentile CT density (Q875)—AUC 0.88 95% CI (0.79 0.94), F1-CT—AUC 0.87 (0.77 0.93) Standard Deviation (SD-CT)—AUC 0.86 (0.73, 0.93). Multivariate models combining CT-density predictors and Neutrophil–Lymphocyte Ratio showed the highest accuracy. SD-CT, Q875 and F1 score were significant predictors of hospital length of stay (LOS) while controlling for hospital death using competing risks models. Moreover, two multivariate Fine-Gray regression models combining the clinical variables: age, NLR, Contrast CT factor with either Q875 or F1 CT-density predictors revealed significant effects for the prediction of LOS incidence in presence of a competing risk (death) and acceptable predictive performances (Bootstrapped C-index 0.74 [0.70 0.78]).

## Introduction

Qualitative and semi-quantitative scoring methods using high resolution computed tomography (HRCT) have been increasingly applied for diagnostic, severity assessment or prognosis of interstitial lung diseases such as idiopathic lung fibrosis (IPF), chronic obstructive pulmonary diseases (COPD), and more recently for severe acute respiratory syndrome (SARS), Middle East Respiratory syndrome (MERS)^1–3^ and COVID-19 pneumonia^4,5^. However, the intra and inter-reader variability remain a substantial limitation. Consequently, alternative objective quantitative methods have been actively explored. Volumetric quantitative CT have been used to predict lung fibrosis outcome^6–8^ for patient stratification or prognosis in COPD, systemic sclerosis, early chronic lung allograft dysfunction in lung transplant patients^9^, ARDS, and recently in COVID-19 patients^10–13^. Two main approaches coexist in Quantitative volumetric CT densitometry: (1) the (“first order”) radiomic method uses the whole lung CT density histogram to extract statistical features such as mean lung attenuation (MLA) standard deviation, skewness and kurtosis, quantile predictors (median, 75th percentile density, etc.) or more advanced features such as entropy^14^. (2) The multi-threshold method uses the whole lung divided in regions of increasing CT density ranges with predefined cutoff values. CT density ranges represent either functional versus non-functional lung regions, or regions associated with emphysema, ground glass opacification, consolidation, etc. Derived features are volumes or volume ratios of different CT density ranges^10,15^.

Both first order radiomic and multi-thresholds methods for CT densitometry have inherent limitations: in the radiomic method, the predefined features mean lung attenuation (MLA), standard deviation, skewness, kurtosis, or entropy were originally defined for simple formal probability distributions, and they are crude descriptors of the often-complex multi-peak CT histograms of the lungs. For example, the kurtosis of a CT density histogram represents both its ‘peakedness’ and the thickness of the histogram left and right tails, making kurtosis hardly interpretable. In the multi-threshold method, multiple non-arbitrary cutoff values are hard to establish. As an example, it took many years to reach a consensus for a single cutoff for emphysema low attenuation area percentage (LAA%) in different CT acquisition conditions^16,17^.

Instead of using predefined formal lung CT density features such as MLA, skewness, or any quantile measurement, it would be ideal to interrogate the whole sample of CT histograms of patient lungs with the disease of interest (e.g., Covid-19) and see how the CT histograms vary in the patient cohort and eventually, which combinations of modes of variation are associated with the severity of the disease or the patient outcome. In the present study, we are using a statistical method called Functional Principal Component Analysis (FPCA) to explore the modes of variation of the lung CT density histograms in Covid-19 patients having either non-enhanced CT or contrast-enhanced CT and to extract new data-driven features for the prediction of patients critical-illness status, hospital length-of-stay and mortality. Performances were compared with a priori methods using CT attenuation quantile values from Q50 (median density) to Q875 (87.5th percentile density), mean lung attenuation (MLA), standard deviation, skewness, and kurtosis.

## Results

### Patient characteristics, clinical, and laboratory findings

Patient characteristics and clinical and laboratory findings are reported in Supplementary Table S1 online. The study included 80 patients (median age 63.5 years, 37 of whom were female). Most patients had at least one comorbidity (71%), from which 30% had 1 comorbidity 49% had 2–3 comorbidities and 21% had 4–5 comorbidities. The mean time between CT and critical illness was 3.7 days (SD 2.7). The mean death time was 24 days (SD 23 days). Critical illness status was defined as meeting one or more of these patient conditions: admission to ICU, requirement for mechanical ventilation, extracorporeal membrane oxygenation (ECMO) or death within 1 month of first presentation to hospital.

The critically ill group included 35 of the 80 patients (44%), with 32 (40%) in ICU, 25 (31%) requiring mechanical ventilation or ECMO and 15 (19%) who died in hospital. More men were critically ill compared to females (23/35, [66%], P = 0.073), with no significant difference in patient age or comorbidities. The critically ill group had a higher mean lactate dehydrogenase value (382 vs 291 U/L, P = 0.003), and a higher mean Neutrophil–Lymphocyte Ratio (16.8 vs 5.71; P = 0.0001).

### CT density curves analysis

**Figure 1:**
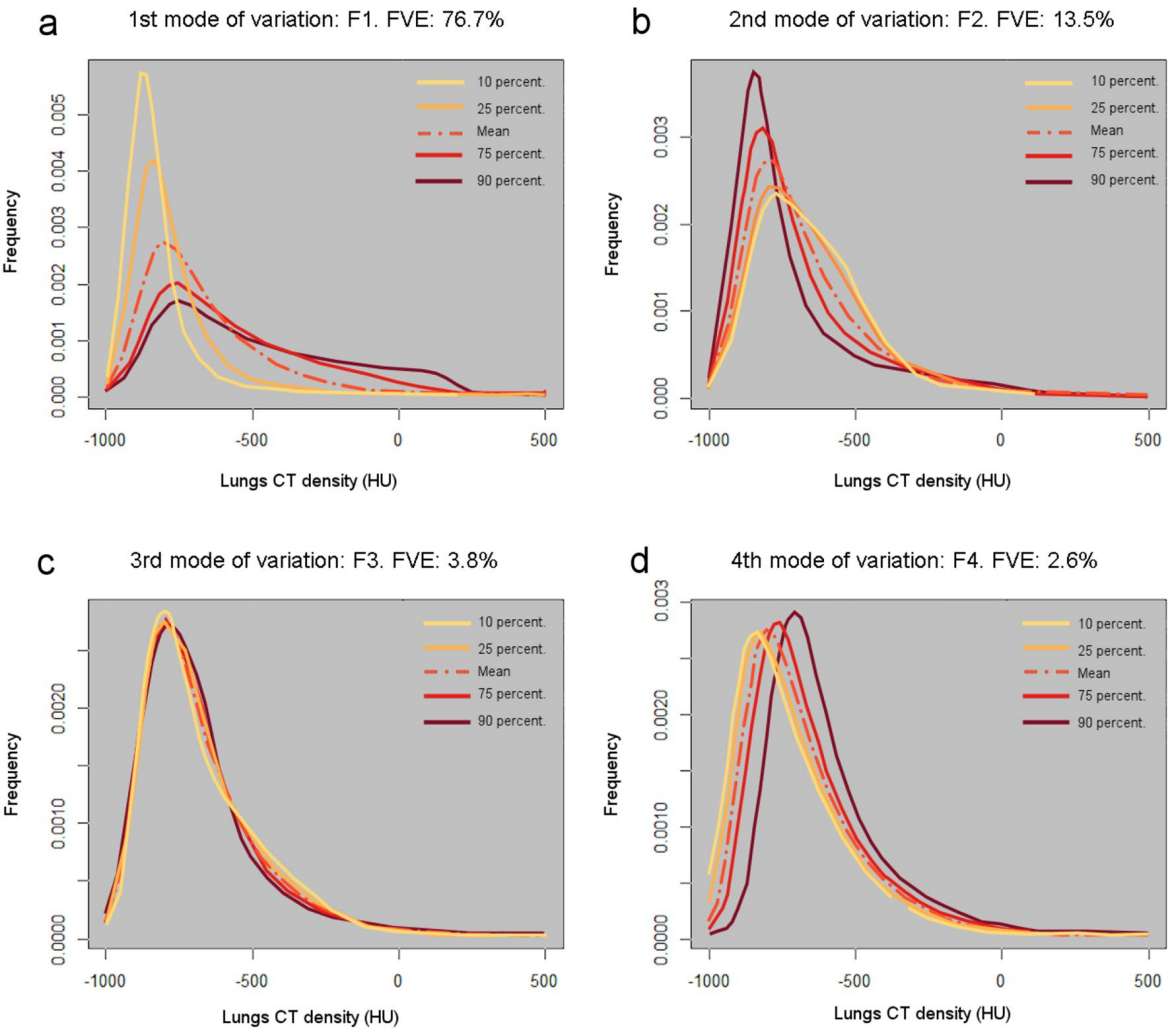
First four modes of variation of the lung CT density curves (smoothed CT histograms). (**a**) F1 explains 76.7% of the fraction of variance explained (FVE). The curves vary from homogeneous low CT density (10 percentile curve) to heterogeneous higher CT density (90 percentile curve). (**b**) F2 explains 13.5% of the FVE. The lung CT density vary from homogeneous low density (90 percentile curve) to include mid-low density in the – 700 HU to – 350 HU range (10 percentile curve). (**c**) F3 explains 3.8% of the FVE. This mode of variation represents a redistribution of CT lung densities from (− 700 HU to – 600 HU) to about (− 600 HU to – 300 HU). (**d**) F4 explains 2.6% of the FVE. F4 represents a small shift of about 140HU from low homogeneous CT densities (10 percentile curve) toward higher CT densities (90 percentile curve).

The Functional Principal Component Analysis (FPCA) resulted in four Functional Principal Components FPCs explaining respectively 76.7% (F1) 13.5% (F2), 3.8% (F3) and 2.6% (F4) of the variability of lung CT density curves in the Covid-19 patients (see Fig. 1). F1 represents the main mode of variation of the lung CT density curves, from homogeneous low CT density (10th percentile curve) consistent with normal lungs density to the heterogeneous distribution of CT density with high densities (above − 100 HU) (90th percentile curve associated with extensive lungs consolidation and ground glass opacification) see Fig. 1a. Two-way Anova shows F1 significantly larger for both the ‘critically-ill’ outcome F(1,76) = 51.2—P < 0.001 and the contrast-CT condition F(1,76) = 6.70—P = 0.012. We expect the high F1 values to be strongly associated with unfavorable outcome (“critically ill” condition) in models stratified by contrast CT conditions.

The second mode of variation F2 represents different degrees of shift in CT density from normal homogeneous low lung CT density (90th percentile curve) toward a larger density range around – 800 HU to – 400 HU (10th percentile curve) see Fig. 1b. Interpretation: high F2 values seem associated with normal lungs while small F2 values seem associated with extensive lungs ground glass opacification but limited consolidation. We expect that the low values of F2 with extensive GGO but no significant consolidation to be moderately associated with unfavorable outcome. The third and fourth modes of variation are associated with small shifts toward higher CT densities: F3 + 46HU (25th percentile curve to 75th percentile curve) and F4 + 61HU (25th percentile curve to 75th percentile curve). See Fig. 1c,d.

Four score values called F1, F2, F3 and F4 quantifying for each patient the lung density curve (once for each mode of variation) were added to the list of existing features for predictive modeling.

### CT density features analysis

The CT analysis findings (including a priori CT features and data driven feature F1) are summarized in Supplementary Table S2 (descriptive statistics), Table 1 (univariate analysis) and Table 2 (multivariate analysis).

**Table 1:** Univariate (ROC) Analysis.

Variables	Unfavorable “critically ill” outcome			
	Cutoff point	AUC (95% CI)	Sensitivity (%)	Specificity (%)
**Best variables—all CT cases (N = 80)**				
Q875 (HU)	> − 380	0.88 (0.79 0.94)	85.7	82.2
F1	> 0.099	0.87 (0.77 0.93)	82.9	82.2
SD-CT (HU)	> 213.8 HU	0.86 (0.73 0.93)	82.86	84.44
**Best variables—contrast CT cases (N = 54)**				
Q875 (HU) C cases (N = 54)	> − 335	0.89 (0.77 0.96)	82.6	87.1
SD-CT (HU) C cases (N = 54)	> 247	0.89 (0.74 0.96)	78.3	96.8
F1 C cases (N = 54)	> 0.330	0.88 (0.77 0.95)	73.9	96.8
**Best variables—non-contrast CT cases (N = 26)**				
Q875 (HU) NC cases (N = 26)	> − 589	0.87 (0.68 0.97)	91.7	78.6
F1	> − 0.255	0.84 (0.64 0.95)	91.7	78.6
SD-CT (HU) NC cases (N = 26)	> 144	0.80 (0.57 0.94)	83.3	71.4
**Other noticeable variables—all cases (N = 80)**				
CT Mean (HU)	> − 646 HU	0.84 [0.73 0.91]	74.29	84.44
Skewness	≤ 1.451	0.83 (0.73 0.90)	88.6	64.4
Kurtosis	≤ 4.680	0.85 (0.75 0.92)	85.7	77.8
Age (years)	≤ 48	0.52 (0.39 0.64)*	34.29	80.00
Lung volume—log (ml)	≤ 3.43 (2670 ml)	0.59 (0.47 0.71)*	74.29	46.67
**Best clinical variable—C & NC cases**				
Neutrophil Lymphocyte ratio C cases (N = 53)**	> 4.9	0.74 (0.60 0.85)	81.82	61.29
Neutrophil Lymphocyte ratio NC cases (N = 25)**	> 4.9	0.77 (0.56 0.91)	81.82	78.57

Best variables (all cases, and cases with and without contrast) and other noticeable variables (Age, Lung volume and neutrophil lymphocyte ratio NLR). Cutoff points were calculated using Youden index. 95% confidence intervals. *Non-significant P value with test on AUC for continuous variables. **1 outlier removed. *NC* non contrast, *C* contrast, *ROC* receiving-operating characteristic.

**Table 2:** Multivariate models for critically ill outcome.

Models for critically ill outcome prediction				
Performance	Accuracy (95% CI)	Sensitivity (%)	Specificity (%)	AUC (95% CI)
**Predictors: CT imaging subjective feature**				
Model 1-N: Best subjective Covid score (Reader 1)—non-enhanced CT cases—N = 26				
Logistic regression	77% (56–91%)	67	86	**0.87 (0.68–0.97)**
Model 1-C: Best subjective Covid score (Reader 1)—enhanced CT cases—N = 54				
Logistic regression	85% (73–93%)	83	87	**0.93 (0.83–0.98)**
**Predictors: CT imaging feature combined with 1 clinical biomarker for critically ill outcome**				
Model 2-N: SD-CT + neutrophil–lymphocyte ratio (NLR)—non-enhanced CT cases—N = 25 (1 outlier for NLR removed)				
Logistic regression	84% (81–87%)	82	86	**0.82 (0.61–0.94)**
Model 2-C: SD-CT + NLR—enhanced CT cases—N = 54				
Logistic regression	87% (85–89%)	86	87	**0.92 (0.81–0.97)**
Model 3-N: F1 + NLR—non-enhanced CT cases—N = 25 (1 outlier for NLR removed)				
Logistic regression	80% (59–93%)	82	79	**0.88 (0.68–0.97)**
Model 3-C: F1 + NLR—enhanced CT cases—N = 54				
Logistic regression	89% (77–96%)	87	90	**0.91 (0.80–0.97)**
Model 4-N: Q875 + NLR—non-enhanced CT cases—N = 25 (1 outlier for NLR removed) NLR is not selected with usual backward selection (P > 0.1)				
Logistic regression	80% (59–93%)	82	79	**0.87 (0.68–0.97)**
Model 4-C: Q875 + NLR—enhanced CT cases—N = 54				
Logistic regression	91% (80–97%)	87	94	**0.92 (0.81–0.98)**

The models with the highest AUC values included: reader-1 subjective Covid score in the enhanced CT group, mean CT density + standard deviation CT density in the enhanced CT group and Standard deviation CT density + NLR in the enhanced CT group. (95% CI = 95% confidence intervals).

Patients with critical illness had significantly different CT density features (all P < 0.0001) with higher CT mean density (MLA), higher CT SD, higher Q875 (87.5th percentile), higher F1 (mean 0.326 vs. − 0.254), lower skewness and lower kurtosis (see Supplementary Table S2 online). Table 1 summarizes all the univariate feature performances (AUC) with optimal cutoff points using the Youden index criteria. Results for the three best CT predictors are stratified into contrast CT and non-contrast CT groups. Notice than for each of these features, the AUCs are slightly higher for the contrast enhanced group than the non-contrast group (non-significant differences). ROC analysis identified as best predictors of critically ill status: Q875—AUC 0.88 (0.79 0.94), F1—AUC 0.87 (0.77 0.93), SD-CT—AUC 0.86 (0.73, 0.93). Remarkably, the frequently used feature trio Mean CT (MLA), skewness and kurtosis showed lower performances than the three previous features: mean CT—AUC 0.84 (0.73 0.91), skewness—AUC 0.83 (0.73 0.90), Kurtosis—AUC 0.84 (0.73, 0.91) and were not retained in the final multivariate models.

Among the clinical variables shown in Table 1, the Neutrophil–Lymphocyte Ratio (NLR) appears the best predictor with AUC 0.74 (0.60 0.85) for the contrast cases and AUC 0.77 (0.56 0.91) for the non-contrast cases and was further successfully included in the final multivariate models. Main univariate ROC curves and AUCs are shown in Supplementary Fig. S1.

### Odds ratios

**Figure 2:**
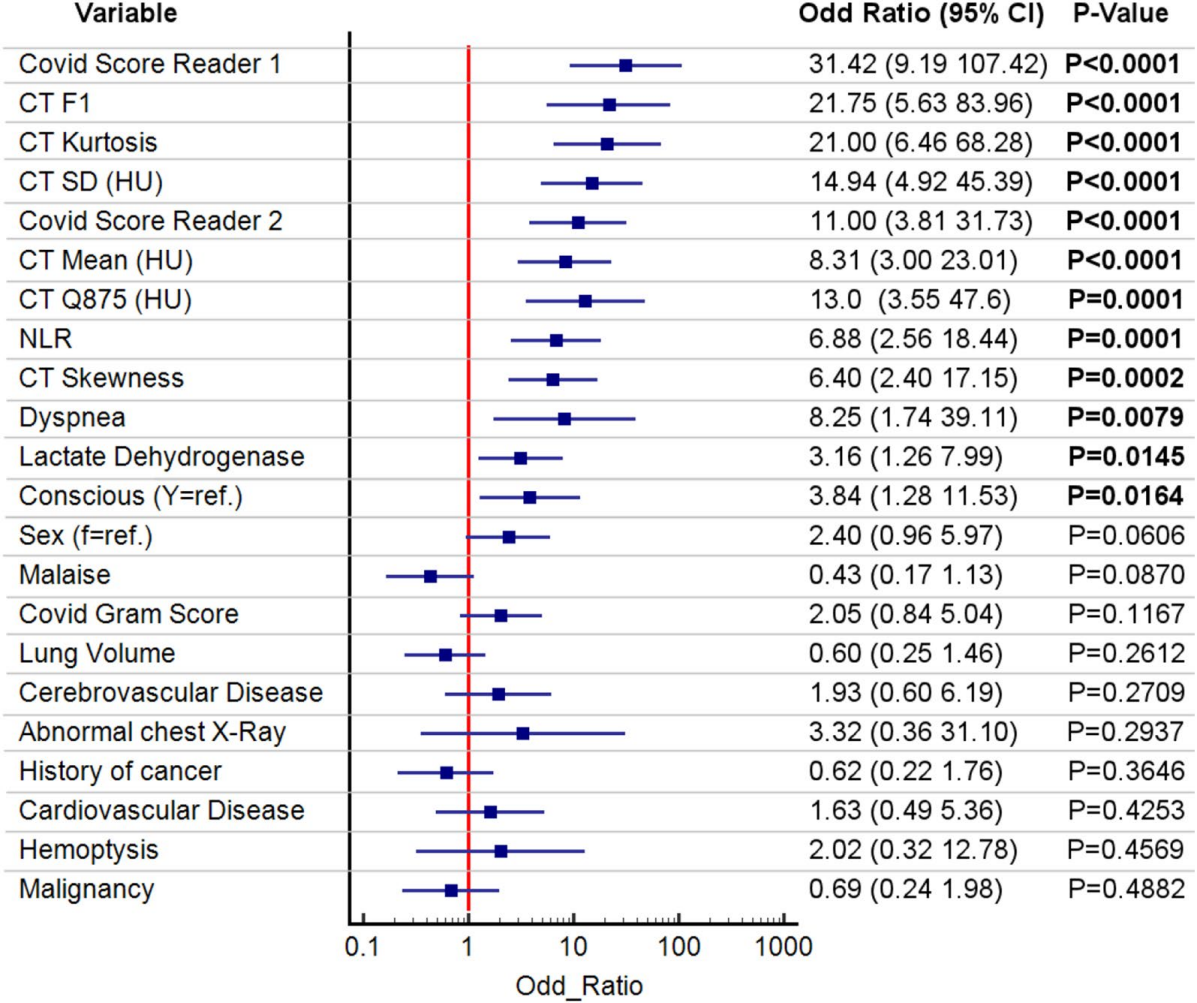
Odd ratios with 95% confidence intervals for critically ill outcome. The continuous variables are split into two groups below / above their median value. Significant P values (P < 0.05) are bold. Variables with P values > 0.5 are not shown. Notice that 6/10 of the highest ranked predictors are lung CT density quantitative features.

Odds ratios (OR) for different quantitative, clinical, and subjective variables are presented in Fig. 2. Whole lungs F1, SD-CT, Mean-CT and Q875 measurements were associated with critical illness: OR from 21.75 (5.63, 83.96) to 8.31 (3.9, 23.1)—all P < 0.0001. CT severity scores had high OR values as well. OR 31.4 (9.2,107.4) P < 0.0001. Neutrophil–Lymphocyte Ratio (NLR) and Lactate dehydrogenase OR values also showed an association with critical illness.

### Multivariate model performances

Using multivariate logistic regression models stratified for IV contrast vs. no contrast to predict critical illness, model 1 with the subjective Covid score alone had the best predictive value: AUC 0.92 (0.83, 0.98) for IV contrast group and AUC 0.87(0.68,0.97) for the non-contrast group. See Table 2. Considering quantitative models, the model 2 combining SD-CT and Neutrophil–Lymphocyte ratio (NLR) predictors had an AUC of 0.92 (0.81, 0.97) with IV contrast compared to AUC 0.82 (0.61, 0.94) the non-contrast group. Model 3 combining F1 and NLR showed an accuracy AUC 0.91 (0.80, 0.97) with IV contrast and AUC 0.88 (0.68, 0.97) for the non-contrast group. The separation of critically ill vs. non-critically ill true cases with the model 3 and its predicted probabilities is shown in Supplementary Fig. S2. Model 4 combining Q875 and NLR predictive accuracy (AUC) showed the highest overall performance among the quantitative models: 0.92 (0.81, 0.98) for the IV contrast group and 0.87 (0.68, 0.97) for the non-contrast group. Separate testing of alternative models using linear and nonlinear classifiers: Linear Discriminant Analysis (LDA), random forests and support vector machines (SVM) did not reveal any improvement of predictive AUCs compared to the selected logistic regression models.

### Combined length-of-stay (LOS) and in-hospital mortality assessment

For SD-CT, Q875 and F1 predictors, the patient cohort was stratified in two groups using optimal cutoff points for combined contrast and non-contrast studies found in the univariate analysis: SD-CT ≥ 213.8 HU, Q875 > − 380 HU and F1 > − 0.099.

**Figure 3:**
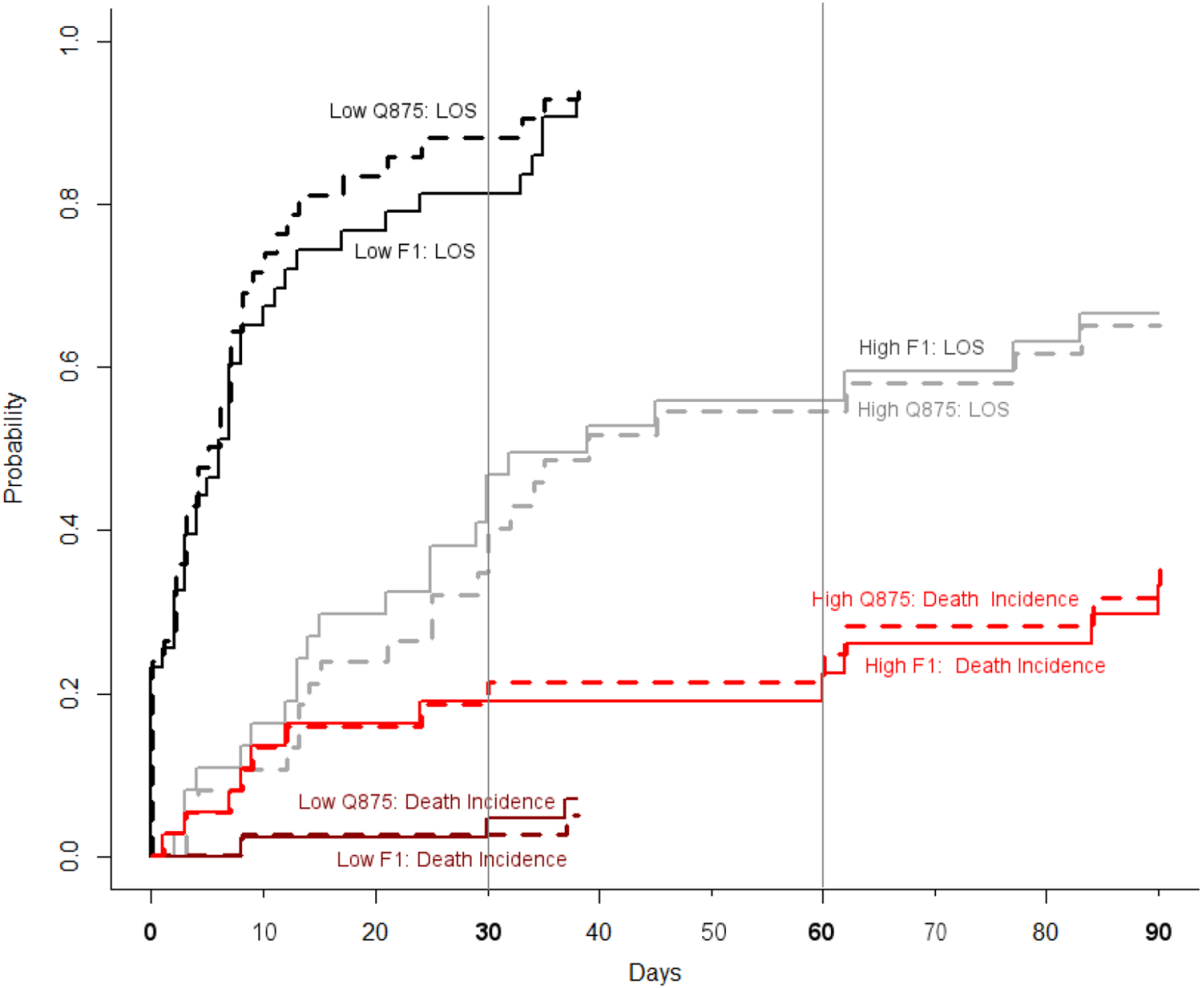
Cumulative incidence plot for hospital length-of-stay (LOS) and in-hospital deaths in Covid-19 patients stratified in Low Q875 (resp. F1) patients and high Q875 (resp. F1) patients using an optimal cutoff point for combined contrast and non-contrast studies found in the univariate analysis: Q875 > − 380 HU (resp. F1 > 0.099). Both F1 and Q875 features give similar predictions of cumulative incidences for LOS and Death during the follow-up period.

Using Q875 feature (see Fig. 3—dashed lines) the cumulative incidence LOS at 30 days was: 89% (80%, 98%) for low Q875 group compared to 40% (24%, 56%) for high Q875 group. the Q875 based cumulative incidence for Death at 30 days was: 2.4% (0%, 7%) for low Q875 group 1 compared to 21% (8%, 34%) for high Q875 group 2. Both inter-group differences in cumulative incidences for LOS (P < 0.0001) and Death (P = 0.027) were significant.

Using F1 feature (Fig. 3—solid lines) the cumulative incidence LOS at 30 days was: 81% (69% 93%) for low F1 group compared to 47% (31% 63%) for high F1 group. The F1-based cumulative incidence for Death at 30 days was: 4.6% (0% 11%) for low F1 group 1 compared to 19% (6% 31%) for high F1 group 2. Inter-group difference in cumulative incidence for LOS (P < 0.0001) was significant while those for Death did not reach significance (P = 0.09).

Similarly, the patients of the low SD-CT group had a probability of being discharged before or at 30 days of 89.7% (80.4% 99.1%) compared to 36.8% (20.9% 52.7%) for the high SD-CT group (P < 0.0001) and a mortality risk at 30 days of 2.3% (0.0% 6.6%) (P = 0.022) compared to 22.4% (8.7% 36.0%) for the high SD-CT group (P = 0.022).

### Multivariate analysis of patient length-of-stay (LOS) with competing risks

The prediction of the hospital length-of-stay (event: hospital discharge) in presence of a competing risk (death) was further investigated in a multivariate framework using the Fine-Gray regression (FGR) for competing risks^18^. Two multivariate models FGR-F1 and FGR-Q875 were selected using the stepwise backward variable selection adapted to the FGR models and further described in the statistical method section. Each of these models included the covariates: Contrast, Age and NLR with either F1 (FGR-F1 model) or Q875 (FGR-Q875 model). See Table 3. For performance comparison, the Reference model (no covariate) and the FGR-1 model without CT density variables (with only Age, NLR and Contrast) were also computed. See Supplementary Fig. S4. Both models FGR-F1 and FGR-Q875 revealed negative coefficients for F1, Q875, Age and NLR indicating that increased values of these covariates would decrease the incidence of the hospital discharge and thus would increase the hospital length of stay. Conversely, in both models the coefficients for Contrast (enhanced CT = 1) were positive, indicating an increased incidence for hospital discharge when the patient got a Contrast CT examination. In other words, the patients with contrast CT had a smaller hospital length-of-stay, all covariates adjusted. See Table 3.

**Table 3:** Multivariate fine-gray regression (FGR) proportional hazards models FGR-F1 and FGR-Q875 for the subdistribution of a competing risk with primary outcome: hospital length-of-stay (LOS) and the competing risk (death).

Variables	Coefficient (Coef.)	exp(coef.)	se(coef.)	z	P values
**Model FGR-F1: LOS ~ age + F1 + NLR + contrast**					
Age	− 0.0187	0.982	0.0067	− 2.78	5.4e−03
F1	− 2.1217	0.120	0.3491	− 6.08	1.2e−09
NLR	− 0.0283	0.972	0.0100	− 2.82	4.8e−03
Contrast:1	0.9807	2.666	0.3081	3.18	1.5e−03
**Model FGR-Q875: LOS ~ age + Q875 + NLR + contrast**					
Age	− 0.0237	0.977	0.006559	− 3.61	3.0e−04
Q875	− 0.0036	0.996	0.000531	− 6.77	1.3e−11
NLR	− 0.0246	0.976	0.009885	− 2.49	1.3e−02
Contrast:1	0.8777	2.405	0.313742	2.80	5.2e−03

All variables in both models have significant effect on the cumulative incidence of the primary outcome (LOS) while controlling for the competing risk (death). Negative coefficients indicate that an increasing value of the variables (age, NLR, F1, Q875) is associated with a decreasing incidence of the hospital discharge (that is, an increasing LOS). Conversely, a positive coefficient (for the Contrast CT factor) indicates an increased incidence of hospital discharge (and reduced LOS).

*exp(Coef.)* exponentiated coefficient, *se(coef.)* standard error of the variable coefficient. N = 80.

Both the FGR-Q875 model and the FGR-F1 model had the same predictive performances: Bootstrap CV C-index 0.74 [0.70 0.78]. Similarly, using the alternative performance metric, the (Bootstrap resampled) Integrated Brier score (IBS) computed on the whole follow-up period: IBS was 0.16 for both FGR-Q875 and FGR-F1 models. For comparison, the IBS—reference model (no covariate) was 0.20 and IBS-FGR-1 model (no CT density covariate) was 0.19. The Supplementary Fig. S4 presents the prediction errors curves for each of these models. Both FGR-F1 and FGR-Q875 models have very similar performances during the 90 days of the follow-up period, confirming in a multivariate setting the similar predictive value of the CT-density variables F1 and Q875 already observed in the univariate analysis (see Fig. 3).

### Semi-quantitative CT severity score and COVID-GRAM scores

The CT severity score was higher in critically ill patients vs. non critically ill patients with a combined mean score amongst both readers of 21 vs 15 (P = 0.002). Univariate AUC was 0.91 (0.80, 0.96) with Reader 1 and AUC 0.83 (0.73 to 0.91) with Reader 2.

The COVID-GRAM score performed poorly with an AUC value of 0.64 (0.52, 0.74). In fact, 79/80 (99%) of our patients were predicted to have medium or high risk for critical illness based on the clinical variables from their medical records compared to the actual value of 35/80 (44%).

### Interrater reliability

Interrater reliability was tested by having a second investigator perform separate Covid CT severity scoring, manual corrections on the lung segmentation (as needed) and measurements on a sample of 20 randomly selected patients.

Interrater reliability for the CT severity score measured with intra-class correlation (ICC) was: 0.90 (0.85 0.94), indicating a good agreement between both readers. Similarly, the quantitative features SD-CT ICC: 0.98 (0.95 0.99), Q875 ICC: 0.99 (0.97 0.99) and F1 ICC: 0.99 (0.96 0.99) showed an excellent interrater agreement.

## Discussion

Parsimonious models combining only one quantitative lung CT density parameter (either SD-CT, Q875, or F1) and one clinical parameter (Neutrophil–Lymphocyte Ratio) allowed accurate prediction of critical illness (ICU, mechanical ventilation/ECMO and/or death) in Covid-19 patients with accuracy (AUC) ranging from 0.82 to 0.92, and prediction of hospital length-of-stay while controlling for the mortality risk. Remarkably, the performances were not adversely affected by the presence of IV contrast in the CT images and were even slightly better in the contrast enhanced group, although the lack of randomization for the IV contrast precludes evaluation of whether this factor leads to better predictions.

The three best CT parameters were Q875, F1 and SD-CT. SD-CT is a well-established a priori radiomic global feature related to the spread of the CT histogram largely available in commercial lung imaging software. In this study, the frequently seen parameters MLA, Skewness and Kurtosis^6^ performed slightly worse than either SD-CT, Q875, or F1 (see Table 1) and were excluded from the final models. Q875 is another radiomic parameter related to the HU value reached when 87.5% of the lung voxels have been counted (starting with the lowest densities). Q875 is the counterpart for the high CT densities of the 15th percentile density index (PD15) used to quantify the severity of emphysema in lung CT densitometry. F1 parameter is a result of the histogram functional principal component analysis (FPCA) in the patient cohort. Without a-priori knowledge or information about the patient outcome, FPCA extracts the main modes of variation in the sample of CT histograms for the patient cohort. F1 (score) values represent the different degrees of CT histograms shift from homogeneous low lung densities (better outcome) toward heterogeneous much higher densities (worse outcome) see Fig. 1. In the current study, other modes of variations (F2, F3, etc.) were not predictive of the patient outcome. F2 seems to reflect the transition from normal lung densities to extended ground glass opacifications (about-800 HU to – 600 HU) and it was not a significant predictor of critically ill status (AUC: 0.53, P:0.60). In this study, the overall results using FPCA are consistent with previous ones for pulmonary disease subtyping^19,20^ or patient neurologic outcome prediction^21^ confirming the value of the FPCA method: first as a non-specific data driven exploration tool, it offers interpretable modes of variations of the CT histograms in the patient whole cohort. Second, it is a generic method giving accurate predictors related to histogram variations without a priori knowledge or delicate radiomic high dimensional parameter selection. All three CT predictors are highly correlated: Spearman Rho Q875 vs. F1: 0.97, Q875 vs. SD-CT, Rho: 0.90, F1 vs. SD-CT: Rho: 0.92 and practically exchangeable. However, the data driven FPCA approach offers a unique data analysis tool of the CT histograms in the whole patient cohort. Current machine learning research is actively extending the FPCA method with supervised FPCA^22^, multivariate FPCA^23^ (neuroimaging data), robust FPCA^24^, etc. offering a rich toolbox for future medical imaging studies. See also Pratt et al.^25^ for a recent application in pulmonology.

The good performances of CT density features for patient outcome prediction in COVID patients are concordant with results from a few previous studies^10–12^ including Lanza et al.^11^ who showed that COVID patients requiring oxygenation and ventilation had higher amounts of compromised lung volumes (− 500 to 100 HU), statistically significant at 6–23% and greater than 23% respectively. Another large study by Colombi et al. showed that a percentage of well aerated lung on CT calculated by software of 71% (OR 3.8, 95% CI 1.9, 7.5, P < 0.001) or less was associated with ICU admission or death^12^.

Lung volume is known to affect the lung CT density in a complex way: first, the optimal lung inflation is difficult to obtain in severe acute lung disease and spirometry-controlled lung CT is often not feasible. So, partially inflated lungs may increase the apparent lung CT density. Bressem et al.^10^ have mentioned the potential confounding effect of the lung volume variation among patients when using CT density as a biomarker in Covid-19 patients. Second, the lung tissue density is increased with disease severity associated with extended GGO and consolidations. Third, lung CT density appears to be lower in subjects with larger lungs because of greater air spaces^26^. In this study, adding the lung volume feature did not improve the performances of the predictive models. However, a moderate but significant correlation has been observed for the CT parameters: Q875—Rho: − 0.60 (− 0.76 − 0.38), F1—Rho: − 0.54 (− 0.72 − 0.30), Mean CT: Rho: − 0.68 (− 0.81 − 0.48) but not SD-CT (Rho: − 0.30 (− 0.49 − 0.08) P = 0.0072, for the non-critically ill patient group, in agreement with the research literature. The supplementary Fig. S3 shows the relationship between Q875 and lung volume for both patient outcome groups. The linear relationship between Q875 and log. Volume in the non-critically ill group and for a large range of lung volumes may be best explained with Robert et al. hypothesis on lung CT density change with normal lung growth^26^.

Moreover, the performance of the clinical predictor: Neutrophil–Lymphocyte Ratio (NLR) in either the univariate analysis (Table 1) or in the multivariate best logistic regression models (Table 2—Models 2–3-4) and multivariate models for LOS prediction (Table 2—Models FGR-875 & FGR-F1) supports the conclusion of the recent meta-analysis from Li et al.^27^ pointing out the value of this biomarker to predict disease severity and patient mortality in Covid-19 patients.

Our quantitative CT density features were compared with both the COVID-GRAM score and CT severity score to predict critical illness. The CT severity score (Reader-1) alone performed well with AUC 0.91 (0.80 0.96) and intra-class correlation (ICC) 0.90 (0.85 0.94) as previously shown in prior studies^4,28^ However, Reader-2 CT severity score was suboptimal and illustrated the inter-reader variability of subjective features. See for example Fig. 2 (odd ratios). The COVID-GRAM score performed poorly in our study to predict critical illness with AUC of 0.64 (0.52, 0.74) 95% CI. A possible explanation of this poor performance compared to the original Chinese study to develop the model by Liang et al.^5^ is the presence of older patients (60.8 vs 48.9 years) and a higher prevalence of one of more pre-existing comorbidities (71.3% vs 25.1%) in our study. Remarkably, Al Hassan et al.^29^ recently reported similar findings with an AUC of 0.64 for COVID-GRAM score for risk stratification with Covid-19 patients.

Hospital length of stay (LOS) and hospital mortality are mutually related and thus require a competing risks method for proper assessment of the cumulative incidence of each event of interest (discharge or death). Using this method, our study showed that the patient groups with Q875 > − 380 HU, F1 > 0.099 or SD-CT > 213.8 HU were all associated with significantly higher cumulative incidences for longer length of stays while controlling for the hospital mortality. Furthermore, our multivariate Fine-Gray models FGR-F1 and FGR-Q875 extend these results combining the significant covariates age, NLR, contrast CT factor and CT-density F1 or Q875 affecting the incidence of the event of interest (LOS) while controlling for the effect of the competing risk (death). Additionally, significant variables found to be associated with a patient outcome (LOS) in our FGR models can be used to develop individual prognostic scores for treatment adaptation. More generally, the prediction of LOS is valuable in capacity planning to provide accurate predictions of the number of beds required at each level of care.

This study has several limitations: it is retrospective and has a modest sample size resulting in larger confidence intervals or suboptimal statistical power when considering subgroup analysis (such as IV contrast vs. Non-Contrast CT) and prevents us to draw conclusions about the in-hospital mortality due to the low number of death events (14/80). The predictive accuracy results were computed with cross-validation correcting performance for overfitting. However, future work involving multiple sites would be necessary for testing the performances in a fully separated testing dataset.

Another limitation is that CT chest protocols varied based on the clinical indication with almost twice as many pulmonary angiogram studies as non-contrast studies, preventing us to better understand the role of CT contrast in the predictive performances.

Finally, the methods discussed in this study are focused on a global lung CT histogram analysis. Multi-threshold lung density analysis methods such as those described in already mentioned studies^10,12,15^ or more advanced CT density/texture methods based on local lung pattern classification^30^ were not tested and should deserve future attention.

In conclusion, the extensive and diffuse changes in lung CT density affecting the whole lungs in COVID-19 pneumonia patients offered the opportunity to compare predefined and data-driven imaging features related to the lungs CT density histograms. All SD-CT, Q875 and F1 features could accurately predict both critical patient illness and hospital length-of-stay. Combined models with one of these features and the biomarker for inflammation Neutrophil–Lymphocyte Ratio gives the highest predictive performance. This application of CT densitometry provided similar results for both Non-enhanced CT group and the contrast enhanced group. The FPCA method allowed the unsupervised analysis of the lung density histograms in the whole patient cohort to extract interpretable CT density features with high predictive values. This approach may be considered for other predictive models with diffuse lung diseases.

## Methods

### Study population

This study was approved by the UHN Coordinated Approval Process for Clinical Research (CAPCR) ethics committee for human research at our home hospital (CAPCR ID: 20-5446). All methods were conducted in accordance with guidelines outlined by this committee. Informed consent was waived due to the retrospective collection of patient data, and this was approved by the CAPCR UHN ethics committee. Inclusion criteria were adult patients ≥ 18 years of age with real-time reverse transcriptase polymerase chain reaction (RT-PCR) confirmed COVID-19 (positive after 1–3 tests) who had undergone a CT chest within 24 h of admission to hospital between March 1 and December 15, 2020 and who were not in the ICU or mechanically ventilated at the time of the CT study. The indication for CT included ruling out suspected COVID-19 with a non-enhanced CT chest and to assess for a pulmonary embolism with an enhanced CT pulmonary angiogram study in patients with confirmed COVID-19. Exclusion criteria were known malignancy with pulmonary nodules on CT (added density), incomplete clinical data and those with a known superimposed bacterial pneumonia. 502 patients were retrieved, with 87 of these patients with confirmed COVID-19 by RT-PCR. Two patients with known lung cancer and lung nodules, three patients with both COVID-19 and a superimposed bacterial pneumonia and two patients with incomplete medical history and blood work were excluded from the study (see Supplementary Fig. S5).

### Patient characteristics on admission

Clinical and laboratory data documented at admission included age, sex, symptoms, blood work (neutrophils, lymphocytes, lactate dehydrogenase, bilirubin) and comorbidities (chronic obstructive pulmonary disease, hypertension, diabetes, coronary disease, heart disease, malignancy, kidney disease, cerebral vascular disease, hepatitis B and immunodeficiency).

### Outcomes

The primary outcome was critical illness, defined as one or more of admission to ICU, requirement for mechanical ventilation or extracorporeal membrane oxygenation and death within 1 month of first presentation to hospital. Secondary outcomes were hospital length of stays and mortality.

### Computed tomography imaging protocol

We analyzed both non-contrast low dose and normal dose CT chest studies and contrast enhanced CT pulmonary angiogram CT studies from three hospitals in our institution. All patients were examined with either 64-CT Aquilion or 320-CT Aquilion-One scanner (Canon Medical Systems, Otawara, Japan). Chest CT acquisitions parameters were 120 kV and 20–100 mA (low dose), 120 kV and 40–150 mA (normal dose) and 100–120 kV and 0–250 mA (contrast enhanced pulmonary angiograms) according to our hospital protocols. All images were reviewed in lung windows (width: 1200 HU, level: − 700 HU) and mediastinum windows (width: 350 HU, level: 40 HU) with 1–3 mm slice thickness. CT pulmonary angiogram studies administered 70 mL of iodinated contrast (Iopromide 370 mg I/mL) at a rate of 5 mL/sec using a bolus tracking technique triggered to 250 HU in the main pulmonary artery.

### COVID Gram score

Clinical and laboratory data at time of hospital presentation was collected from the patients’ electronic medical records to calculate the COVID-GRAM score^5^. These 10 parameters include age, dyspnea, conscious, hemoptysis, history of malignancy, number of comorbidities, X-ray abnormality, Neutrophil–Lymphocyte Ratio, lactate dehydrogenase and direct bilirubin. An online calculator was used to calculate a risk score and percentage and place the patient in a low, medium, or high-risk group to predict critical illness^31^.

### CT severity score

Subjective assessment of the percentage of ground glass opacities and consolidations on CT chest was performed by two radiologists, one with 4 years of clinical experience as a staff thoracic radiologist, and one a cardiothoracic imaging fellow with 5 years of radiology residency experience. The lungs were evaluated as per the CT Severity Score guidelines^4^ by assigning a score of 0–2 (0 = no opacity, 1 =  < 50% opacity and 2 =  ≥ 50% opacity) in each of the 10 segments in both lungs out of a total score of 40.

### CT density analysis

**Figure 4:**
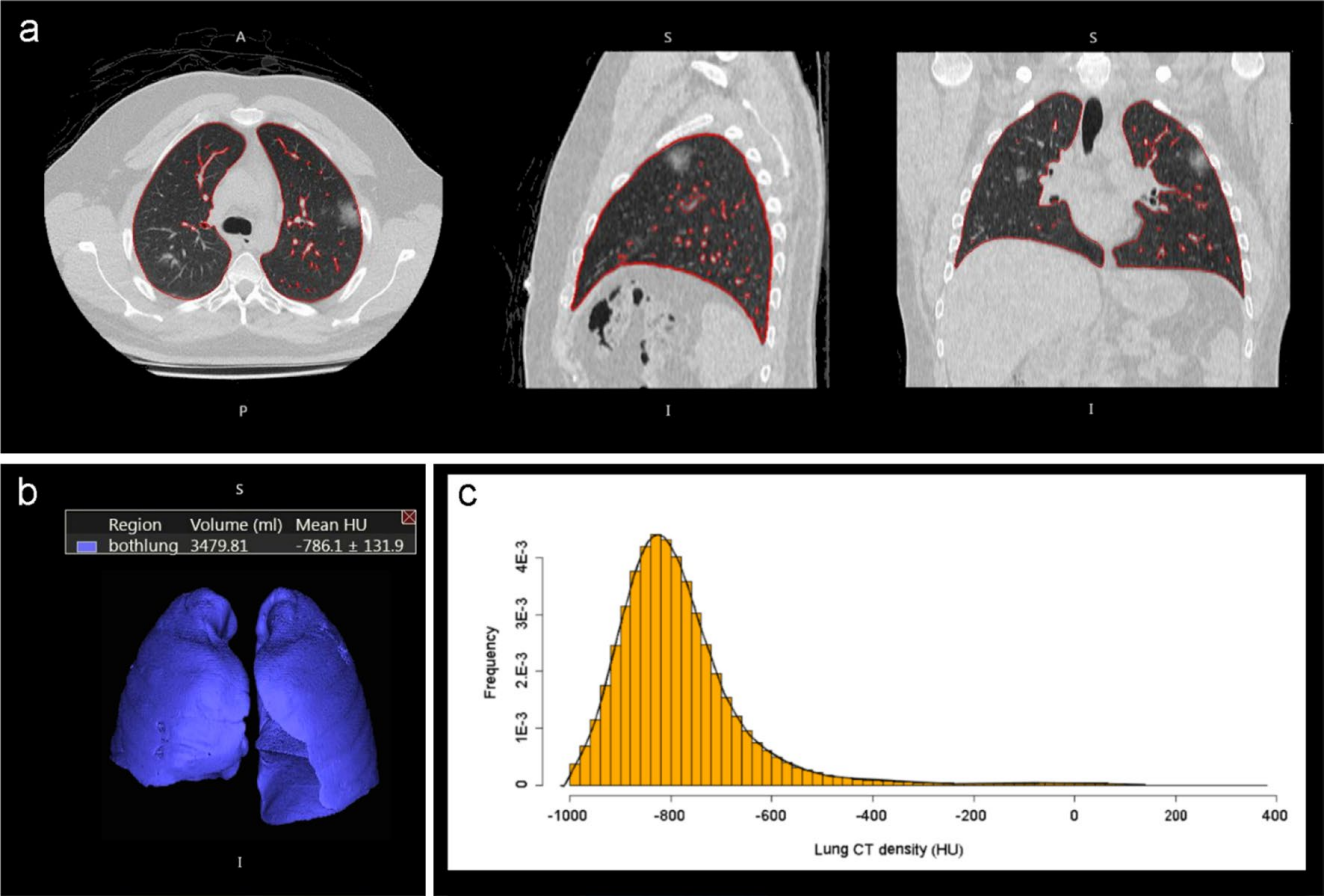
Non-contrast chest CT images in a 28-year-old man with mild COVID-19 pneumonia. The patient was discharged 3 days later without ventilation or ICU admission. (**a**) CT images show small, rounded ground-glass opacities in the upper lobes. (**b**) 3D view with volume. (**c**) Histogram of CT density values for both lungs shows most lung voxels in a normal lung density distribution and peak at about − 800 HU. (perc. = percentile). Mean lung CT density: − 786.1HU (21st perc.), SD-CT: 131.9HU (29th perc.), Skewness: 2.58 (97th perc.), Kurtosis: 13.40 (92nd perc.), Q875: − 664HU (19th perc.), F1: − 0.43 (19th perc.), F2: -0.05 (39th perc.).

**Figure 5:**
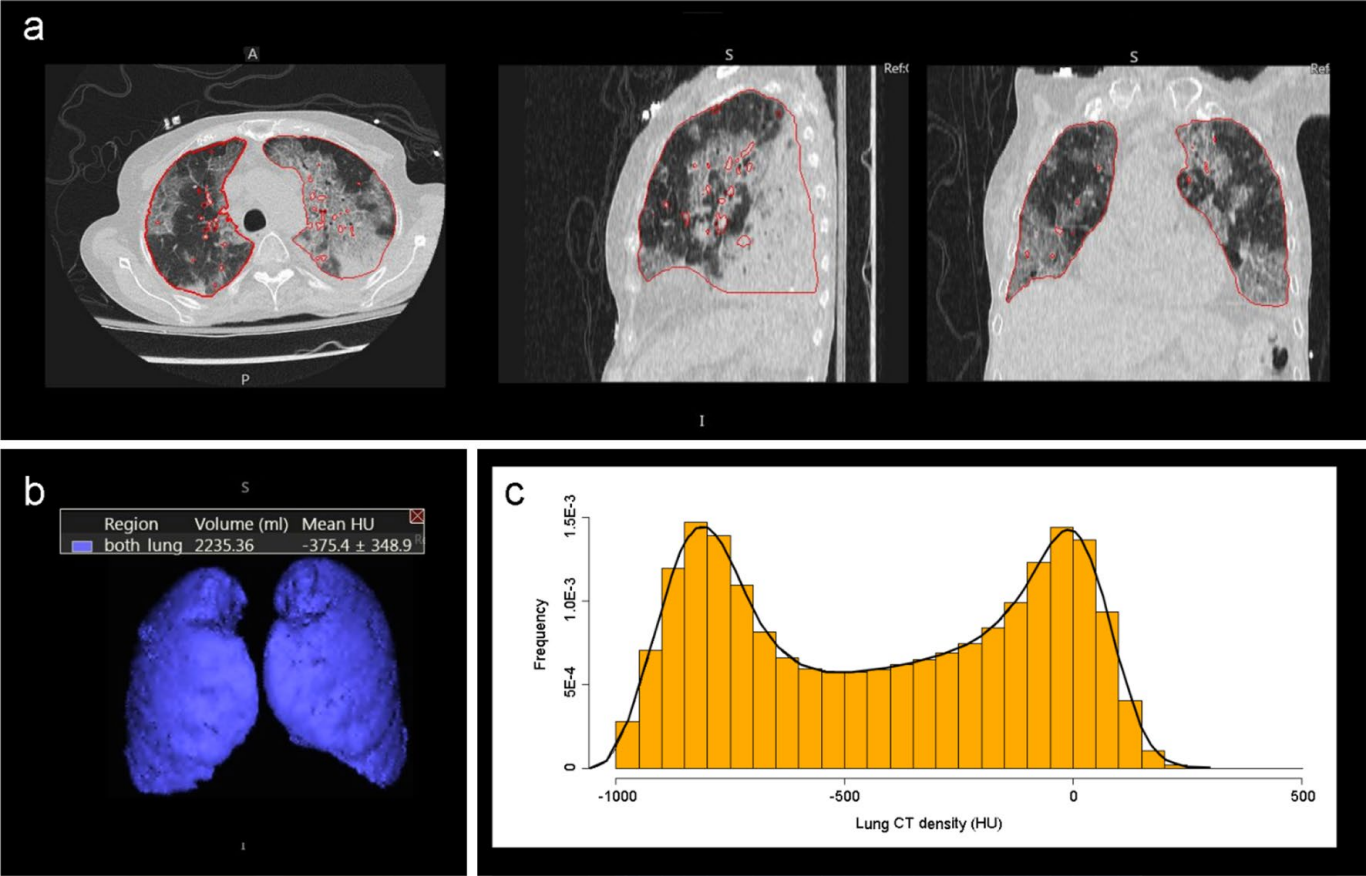
Non-contrast low dose chest CT images in a 62-year-old woman with severe COVID-19 pneumonia. The patient was admitted to ICU, required ventilation, and died in hospital. (**a**) CT images show bilateral extensive ground glass opacities and septal thickening. (**b**) 3D view with volume. (**c**) Histogram of CT density values demonstrates 2 peaks, the first peak at − 800 HU representing normal well aerated lung and the second peak at 0 HU representing the lung consolidations and ground glass opacities. Mean lung density: − 375.4 HU (98th percent.), SD-CT: 348.9 HU (94th perc.), skewness: − 0.02 (7th perc.), kurtosis: 1.53 (0.6th perc.), Q875: 36HU (88th perc.), F1: 0.77 (98th perc.), F2: − 0.33 (9th perc.).

Lung density measurements were performed using Vitrea Advanced Visualization version 7.14 (Canon Medical, Minnetonka, USA) software to perform automatic lung segmentation and calculate a mean CT density (Mean-CT) and standard deviation (SD-CT) density for both lungs combined (see Figs. 4, 5). Large pulmonary vessels, airways, mediastinum structures and pleural effusions are excluded from segmentation, and lung parenchyma, interstitial structures and segmental vessels and bronchi were included. Manual correction of the lung segmentation was applied when needed. The primary density analysis was performed by a cardiothoracic radiology fellow, and a second analysis to determine inter-rater reliability on a random sample of 20 cases from the data set was performed by a clinical research analyst with 4 years of experience in cardiothoracic radiology.

### CT density curves analysis

Each CT density histogram for both lungs and each lung were converted in smooth curves defined between – 1000 HU and 500 HU using Ramsey’s smoothing method for frequency distributions^32^ and previously described^21^. Quantiles values on the CT histograms from median (50th percentile) to 85.7th percentile were computed and added to the feature list. A Functional Principal Component Analysis (FPCA) was applied to the lung CT density curves following Petersen & Müller’s method for frequency distributions^33^. FPCA is a data driven approach akin to the Principal Component analysis (PCA) to explore and quantify the main modes of variation of a sample of curves. The resulting functional principal component scores (FPCs) for the lung region were added to the list of candidate predictors of the patient outcome including lung volume, demographic information and a priori CT density-based features: mean lung CT attenuation, standard deviation, skewness, kurtosis and eight quantile-based features.

### Statistical analysis

Statistical analysis was performed using R statistical programming and MedCalc software. A P value of < 0.05 was considered statistically significant. Continuous variables were described using mean and standard deviation or median and interquartile range and categorical variables using numbers and percentage. Mann–Whitney tests and Fisher exact tests were used to compare continuous and binary variables. Variance ratio F-test were used to compare inter-group variance differences. Inter-rater agreement was assessed using intra-class correlation (ICC). Logistic regression models were applied for prediction of critical illness. A univariate analysis of the predictors for critical illness was performed using a receiver operating characteristic (ROC) area under the curve (AUC) metric for continuous predictors and odds ratio (OR) for binary predictors. First quartile to third quartile differences were used for defining OR in non-categorical variables. Optimal cutoff points on ROC curves were determined using the Youden index method.

In the multivariate analysis, predictive accuracy (AUC) of the final logistic regression models were corrected for overfitting using a Bootstrap cross-validation method. Backward variable selection was performed separately for models with qualitative assessment variables (subjective reader scores) and those with quantitative CT Imaging feature combined with 1 clinical biomarker for critically ill outcome. Highly correlated CT density predictors SD-CT, Q875 and F1 were tested in separate models. See Table 2. Final model selection was based on Bootstrap overfitting corrected AUC.

The length-of-stay estimate for Covid patients have recently been addressed with numerous methods^34^. In this study, the hospital discharge time was used as primary end point for hospital length-of-stay (LOS) and the in-hospital death was considered as the competing risk, following Brock et al. approach and publicly available R code^35^. A cumulative incidence plot of hospital discharge was computed in R-programming language using the Aalen-Johansen estimator. The Greenwood-type method was used to estimate the standard errors and confidence intervals of our cumulative incidence plots. The prediction of the hospital length-of-stay (event: hospital discharge) in presence of a competing risk (death) was further investigated in a multivariate framework using the Fine-Gray regression (FGR) for competing risks^18^. FGR applies a proportional hazards model for the direct estimation of the hazard of the cumulative incidence function (CIF) or subdistribution for the primary event (hospital discharge). Quantitative variables of interest, that is, those with significant odd ratio for the ICU prediction (see Fig. 2—bold font) with the age and the CT Contrast factor were combined for a stepwise variable selection using the R-library ‘crrstep’^36^. The Bayesian Information Criterion BICcr was used for model selection. BICcr is a modified version of BIC adapted for FGR in using the total number of (uncensored) primary events (since only these events contribute to information of the partial likelihood) as a penalty and allows for a more parsimonious model than AIC but with a less stringent penalty than BIC^36^. The performance of the Fine-Gray Regression models FGR-F1 and FGR-Q875 for the prediction of the hospital discharge (or equivalently the LOS) was evaluated using both Concordance index (C-index) and Brier score. The Brier score is a weighted average of the squared distances between the observed survival status and the predicted survival probability of a model. Performance assessments were computed with the R package ‘PEC’^37^.

## Supplementary Information

Supplementary Information.

## Data Availability

The R-code for the CT density histogram smoothing and for the functional principal component analysis used in the current study are available from the corresponding author.
