# Supplementary material for: Predefined and data driven CT densitometric features predict critical illness and hospital length of stay in COVID-19 patients
Source: Sci Rep. 2022 May 17;12:8143. doi: 10.1038/s41598-022-12311-4 (PMC9114017; doi:10.1038/s41598-022-12311-4)
Supplement: Supplementary file 1 — Supplementary Information. [file 41598_2022_12311_MOESM1_ESM.docx]

**Supplementary Information**

**Predefined and data driven CT densitometric features predict critical illness and hospital length of stay in COVID-19 patients**

Tamar Shalmon MD^1,2^, Pascal Salazar PhD^3^, Miho Horie PhD^1,2^, Kate Hanneman MD, MPH, FRCPC^1,2^, Mini Pakkal MD, FRCR^1,2^, Vahid Anwari MRT^1,2^ and Jennifer Fratesi MD, FRCPC^1,2^ *

^1^ Joint Department of Medical Imaging, University of Toronto

^2^ University Health Network, Toronto General Hospital, Toronto, ON, 200 Elizabeth St, Toronto, ON, M5G 2C4

^3^ Vital Images, Minnetonka, Minnesota, USA

* Corresponding Author: [Jennifer.fratesi@uhn.ca](mailto:Jennifer.fratesi@uhn.ca), University Health Network, Toronto General Hospital

**Supplementary Table S1. Patient characteristics and outcomes.**

Median (1st quartile, 3^rd^ quartile value), mean and p-values. Tests for differences: Man-Whitney test (continuous values) and exact Fisher test (nominal variables). Bold P-values are significant. ECMO=extracorporeal membrane oxygenation

| Characteristic Value: Median (25^th^perc. 75^th^perc.). Mean. Count if categorical | All cases  (N=80) | Group 1  Not Critically Ill  (N=45) | Group 2  Critically ill  (N=35) | Difference Test P-value |
| --- | --- | --- | --- | --- |
| **Demographic, condition and CT Acquisition** | | | | |
| Age (years) | 63.5 (48 74.2)  Mean: 60.7 | 63.0 (51 74)  Mean: 61.4 | 64.0 (45 74.5)  Mean: 60 | P=0.73 |
| Sex | M: 43 F: 37 | M: 20 F: 25 | M: 23 F: 12 | **P = 0.073** |
| Conscious | Y: 61 N: 19 | Y: 39 N:6 | Y: 22 N: 13 | **P= 0.018** |
| Dyspnea | Y: 63 N: 17 | Y: 30 N: 15 | Y: 33 N: 2 | **P=0.003** |
| Comorbidities (10)  COPD  Hypertension  Diabetes  Coronary heart disease  Chronic heart disease  Chronic kidney disease  Malignancy  Cerebral vascular disease  Hepatitis B  Immunodeficiency | 0: 23  1: 17  2-3: 28  4-5: 12 | 0: 15  1: 7  2-3: 16  4-5: 7 | 0: 8  1: 10  2-3: 12  4-5: 5 | P=0.50 |
| Lactate Dehydrogenase (U/L) | 273 (217 402)  Mean: 331 | 250 (198 328) Mean: 291 | 357 (248 476) Mean: 382 | **P=0.003** |
| Neutrophil Lymphocyte Ratio | 5.0 (3.0 10.2)  Mean: 10.6 | 4.1 (2.7 5.2) Mean: 5.71 | 9.6 (5.0 14.8) Mean: 16.8 | **P= 0.0001** |
| Contrast-enhanced CT  C=Contrast ; NC= Non-Contrast | C: 54, NC: 26 | C: 31 NC: 14 | C: 23 NC: 12 | P = 0.810 |
| **Outcome** | | | | |
| Critically ill | Y: 35. N: 45 | - | Y: 35 N: 0 | - |
| ICU | Y: 32. N: 48 | - | Y: 32 N: 3 | - |
| Mechanical Ventilation/ECMO | Y: 25. N: 55 | - | Y: 25 N: 10 | - |
| Death | Y: 15. N: 65 | - | Y: 15 N: 20 | - |
| Days in hospital | 9 (3 30)  Mean: 18 | 4 (2 9)  Mean: 8 | 29 (13 43)  Mean: 33 | **P<0.0001** |

**Supplementary Table S2. Quantitative CT related measurements and CT Severity Scores**

Median (1st quartile, 3^rd^ quartile value), mean and p-values. Tests for differences: Man-Whitney test (continuous values) and exact Fisher test (nominal variables). Bold P-values are significant. Patients in the critically ill group had significantly higher mean density and standard deviation density values in both lungs combined and noticeable but not statistically significant lower lung volumes. Subjective scoring was significantly higher in the critically ill group for both readers.

| Quantitative and Subjective CT variables: Median (25^th^perc. 75^th^perc.) Mean. Count if categorical | All cases  N=80 | Group 1  Not Critically Ill  (N=45) | Group 2  Critically ill  (N=35) | Difference Test P-value |
| --- | --- | --- | --- | --- |
| CT Mean (HU) | -664 (-773 -550) Mean: -629 | -720 (-785 -657) Mean: -721 | -549 (-651 -391) Mean: -512 | **P < 0.0001** |
| CT Standard Deviation (HU) | 204 (137 257) Mean: 207 | 168 (118 203) Mean: 164 | 261 (225 303) Mean: 262 | **P < 0.0001** |
| CT Skewness | 1.27 (0.76 1.96)  Mean: 1.28 | 1.87 (1.10 2.170  Mean: 1.69 | 0.77 (0.37 1.28)  Mean: 0.76 | **P < 0.0001** |
| CT Kurtosis | 4.74 (2.98 9.17)  Mean: 6.22 | 8.11 (4.87 10.66)  Mean: 8.13 | 2.98 (2.30 4.18) | **P < 0.0001** |
| Q875 (HU) | -425 (-640 -126)  Mean: -379) | -588 (-669 -446)  Mean: -546 | -55 (-332 24)  Mean: -164 | **P < 0.0001** |
| F1 | 0.047 (-0.383 0.395)  Mean: 0.00 | -0.324 (-0.449 -0.057)  Mean: -0.254 | 0.421 (0.153 0.604)  Mean: 0.326 | **P < 0.0001** |
| Lung volume (ml) | 2555 (1971 3315)  Mean: 2799 | 2643 (1964 3449)  Mean: 3001 | 2305 (1980 2984)  Mean: 2540 | P=0.1582 |
| Subjective scoring | | | | |
| Covid Score Reader 1 | 20 (12 26.25) Mean: 19.55 | 13 (8 19) Mean: 13.4 | 27 (23 32)  Mean: 27.5 | **P < 0.0001** |
| Covid Score Reader 2 | 20 (12 26.25) Mean: 19.55 | 12 (7 19)  Mean: 13.9 | 26 (20.5 33) Mean: 25.8 | **P < 0.0001** |
| Covid Score Mean Reader 1-2 | 16.25 (11.9 24) Mean: 17.5 | 14.5 (10 21) Mean: 15.0. | 20.7 (14.25 26.5)  Mean: 20.7 | **P = 0.0020** |

***Supplementary Figure S1*. Receiver Operating Characteristic (ROC) curves and Area-Under-the-Curves (AUCs) for CT severity score (reader-1), F1, Q875, standard deviation - CT density and COVID gram score.** The gray diagonal line represents the random classifier line. All cases (N=80)


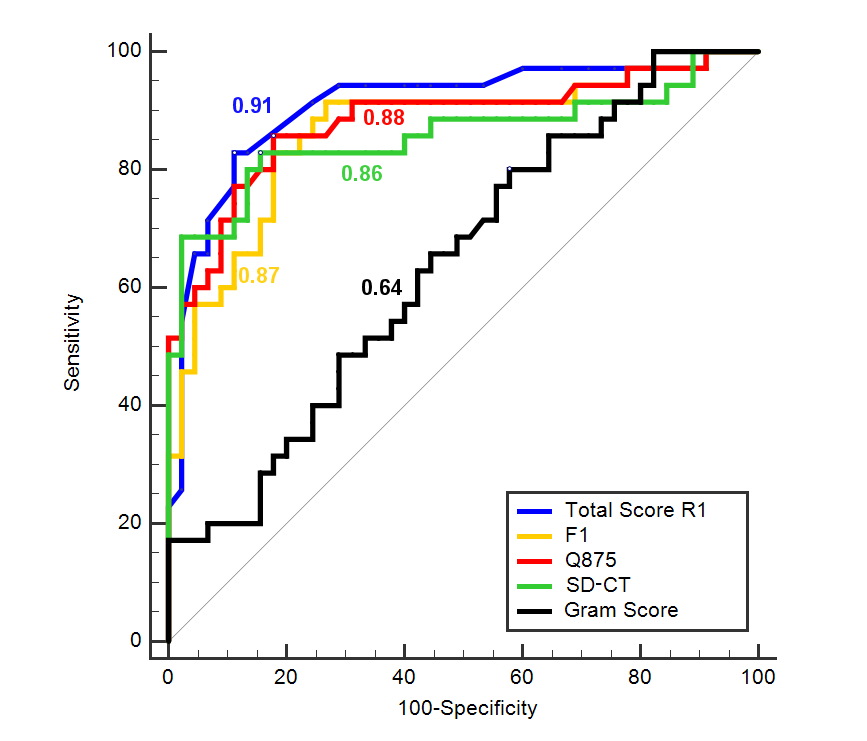


***Supplementary Figure S2*. F1 values vs. Neutrophil-Lymphocyte Ratio (log.10 NLR – centered) with true critical Illness cases: C1 (black triangles) and true non-critical illness cases: C0 (white dots).** N=79 (1 NLR outlier removed). Overlay color and lines: estimated probabilities of critical illness (C1) from logistic regression model 3 (combining F1 and NLR). This bivariate linear model provides a clear separation for critical illness outcome on each side of the p=0.50 midline (yellow line).


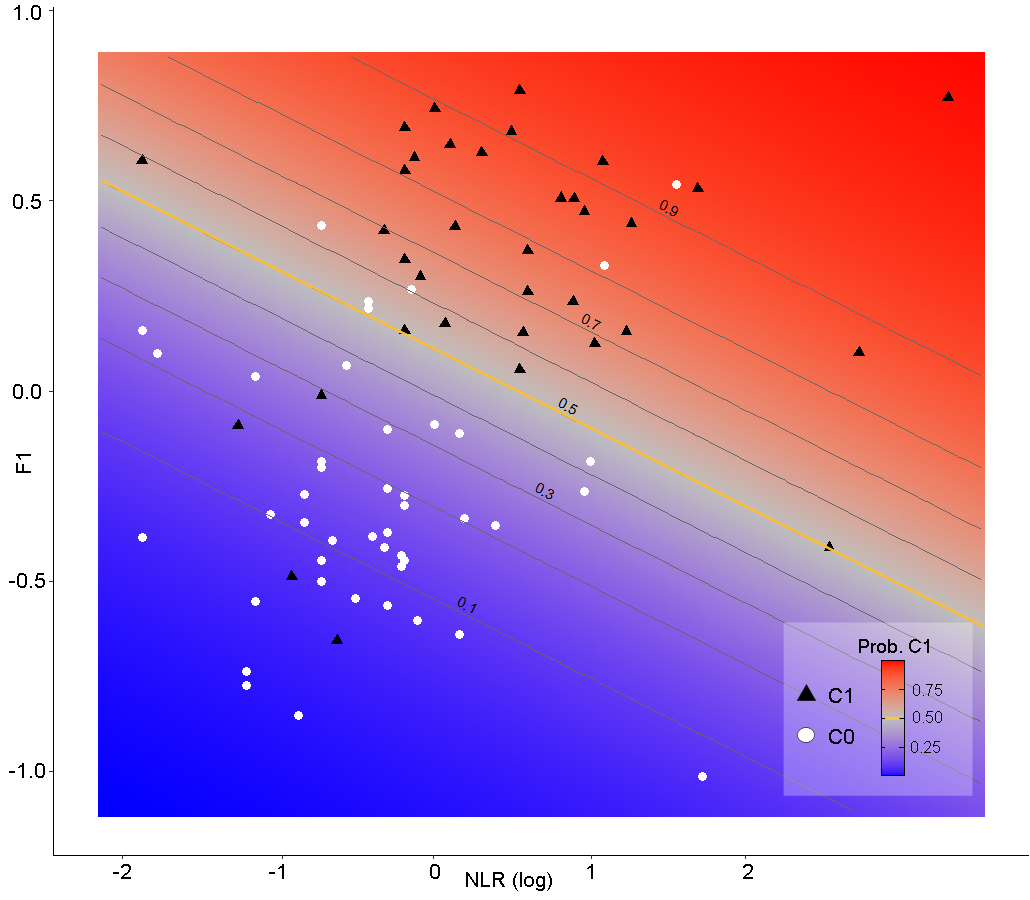


***Supplementary Figure S3*. Scatterplot Q875 values vs. lung volume (log scale) and Spearman rank correlations (Rho)** – C1: Critically ill group. C0: Non-critically ill group. N=80. The linear relationship between Q875 CT related predictor and the lung volume is clearly visible for the C0 non-critically ill group. Lines: local regression smoothing (LOESS) trend lines.

**
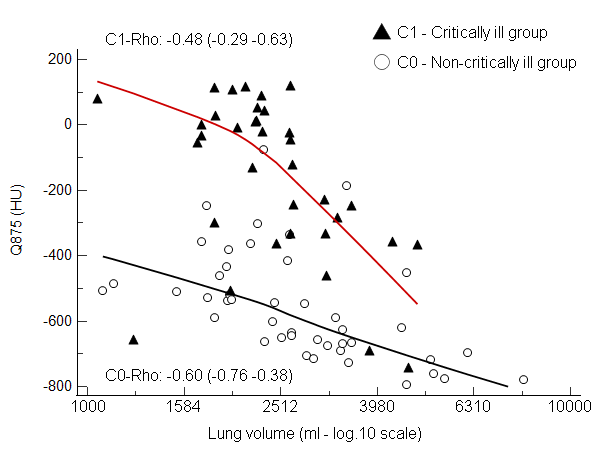
**

***Supplementary Figure S4*. Prediction Error Curves for Fine-Gray Regression models: FGR-1, FGR-875, FGR-F1 and the Reference Model (‘No Covariate’ model)**. Prediction error curves are obtained when the Brier score is followed over time - All patients (N=80). Both multivariate models combining CT-density variables FGR-F1 and FGR-Q875 with clinical variables Age, NLR and the CT Contrast factor have the lowest prediction errors. FGR-1 model only includes the clinical parameters Age, NLR and the CT Contrast factor.


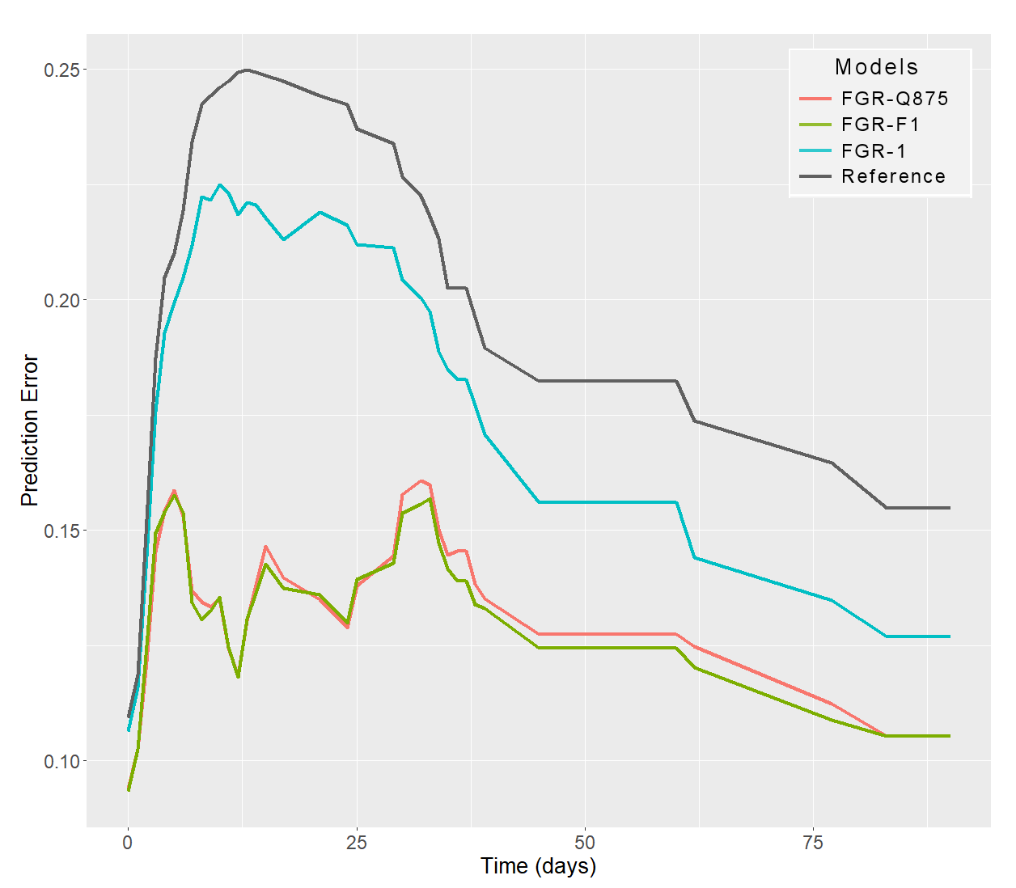


***Supplementary Figure S5*. Patient flow chart with inclusion and exclusion criteria for patient selection in the study.**

87 patients had a positive RT-PCR test for COVID-19 after 1-3 tests

502 patients appeared in PACS search engine when searching for COVID and CT chest between March 1 and December 15, 2020

415 patients had a negative RT-PCR after 1-3 tests

85 patients did not have lung nodules/masses in their lungs on CT chest

2 patients had a history of cancer with lung nodules or masses on their CT chest

82 patients did not have sputum proven superimposed bacterial pneumonia known during their hospital stay

3 patients had a sputum test during their hospital stay showing superimposed bacterial pneumonia

80 patients had complete demographic and laboratory information in their electronic patient records

2 patients had incomplete information in their electronic patient records
